# The Principles and Practice of Midwifery. In Which Are Comprized and Methodically Arranged under the Four General Heads of Generation, Gestation, Delivery, and Recovery, All the Anatomical Facts, Physiological Reasonings, Pathological Observations and Practical Precepts, Necessary to Constitute the Fullest and Most Complete System of Midwifery

**Published:** 1781-06

**Authors:** 


II. *The Principles and Practice of Midwifery. In which are comprized and methodically arranged under the four general heads of Generation, Gestation, Delivery, and Recovery, all the anatomical facts, physiological reasonings, pathological observations and practical precepts, necessary to constitute the fullest and most complete system of Midwifery. By* Edward Foster, M. D. *late Teacher of Midwifery in the City of Dublin. Completed and corrected by* James Sims, M. D. 8vo. Baldwin, *London*, 1781, 316 *pages*, 4s. *in boards.*

THE performance here offered to the public, was intended, it seems, by the author, as a text-book to his lectures; yet at the same time to be plain and intelligible to those who might not have the advantage of hearing his own comment. Unfortunately, however, he was cut off by a fever in the prime of life, before he had finished or revised the work. The task of correcting and compleating it was undertaken by Dr. Sims, who appears to have been actuated by a laudable zeal for the reputation of his deceased friend.

The work is composed in the form of aphorisms; a mode of writing, as the author himself very very properly obſerves, which precludes the introduction of diffuſe arguments, or a ſplendid ſtyle. He ventures, however, to recommend his performance "not only to the ſtudent, but to the "practitioner, as what has borne the teſt of ex-"periment, and thence likely to endure the "tooth of time."

The matter is arranged under the four general heads enumerated in the title. The firſt, on generation, contains a conciſe but accurate deſcription of the female organs of generation; together with the ſymptoms and general curative indications of the diſeaſes they are liable to. The author likewiſe takes occaſion to relate the different theories of menſtruation and conception. The former of theſe he aſcribes to, 1. the peculiar ſtructure of the uterus; 2. to a general plethora; 3. to a vaſcular ſpaſm, produced by the two former cauſes, and always demonſtrable by the pulſe.

"Conception, he ſays, occurs in the ovaria, "by the communication of the male ſemen to "them through the womb and Fallopian tubes, "and by its abſorption into a mature ovum; "where, by mixture with the liquor of this "ovum, it forms the rudiments of the em-"bryo."

Speaking of præternatural formation, he very justly denies that it can be owing to any powers in the imagination of the mother, who is generally ignorant when, and always how, conception happens. Befides, as the author obferves, her imagination poffeffes no fuch power over any part of her own body; there is no communication by blood veffels or nerves between the mother and fœtus; præternatural formation often appears without any emotion in the mother's imagination, which on the other hand is frequently difturbed without any fuch effect taking place; and in fine, a deviation from the natural form is obferved occafionally to happen, not only in every fpecies of animals, oviparous as well as viviparous, but alfo in vegetables. He is therefore induced to afcribe it to "imperfection or difproportion in the prolific "principles of male or female, or of both, "producing fuperfluity, deficiency, or difproportion;" 2. to "a difturbed union of thefe "principles producing disfiguration and diflocation;" and 3. to "injury received externally, by contufion, compreffion, &c. or internally, by difeafe, producing concretion or "deficiency."

In the fecond part of the work, which treats of of geſtation, we are preſented with an account of the alteration that takes place in the bulk, ſhape, and ſituation of the uterus; the ſize, weight, and poſition of the fœtus at different periods, &c. He is of opinion, that ſome ſhare of its nutriment is evidently derived from the liquor amnii; and in deſcribing the involucra, he ſpeaks of the external chorion as being "the "*membrana decidua* of a celebrated contempo-"rary anatomiſt."

Among a variety of other ſymptoms of uterine geſtation, the author mentions a diſlike of coition, paleneſs, and ſometimes a tawny colour, chiefly around the eyes; alſo a curvature of the ſpine and body backwards, to preſerve the centre of gravity on the thighs, with a conſequent waddling in the gait, and propenſity to fall.

In treating of extra-uterine geſtation, the author obſerves, that the womb, if examined by the touch, is found in the unimpregnated ſtate; and indeed he is not ſingular in this, as it has been the generally received opinion; but in a female ſubject lately diſſected in London, where the fœtus and ſecundines had been contained in one of the Fallopian tubes, the uterus was found to have increaſed in the ſame proportion as in natural pregnancy.

In

In the therapeutic part of geſtation, the author notices the various diſeaſes attendant on that ſtate, together with the methods of treating them.

The third part treats of delivery, which the author divides into natural and præternatural. The former of theſe he aſcribes to an irritation of the womb, ariſing ſeemingly from that dilatation of its orifice, which muſt always take place ſo ſoon as the cavity of the collum uteri is completely diſtended by geſtation.

Præternatural labour, he thinks, is reducible to four heads, viz. "1. ſlow; 2. inſtrumental; "3. wrong preſentations; and 4. complex." The various cauſes of each of theſe, and the methods of remedying them, are accurately pointed out.

In the caſe of twins, we are adviſed to truſt the delivery of the ſecond fœtus to nature, unleſs the ſymptoms ſhould be ſuch as require aſſiſtance. In theſe caſes we are told, that "after a ſhort reſpite of ſome minutes, or perhaps ſeldom of ſome hours, and very rarely "of a few days, natural labour returning expels the ſucceeding fœtus, even more ſpeedily "and eaſily than the former: Nature (adds "the author) requires only our official, not

"officious aid." Speaking of the rupture of the uterus from too great violence uſed in turning, the author remarks, that it "has ſometimes, though rarely happened, that the fœtus "and ſecundines being propelled into the cavity of the abdomen, and the womb contracting, which it generally ſoon does, even in "fatal caſes, to a very moderate ſize, the woman has ſurvived and carried about this load "of extra-uterine fœtus."

In the fourth and laſt part of his work, the author treats of recovery, or "the tranſition to "the ſound from the infirm ſtate, conſequent "to delivery," which he divides into natural and præternatural. He obſerves, that "when "the coloſtrum and milk are duly evacuated, "there is no ſuch fever as authors have conſtantly deſcribed to be neceſſary to the commencement of this ſecretion, about the third "or fourth day after delivery."

The diſeaſe that is found moſt frequently to retard or prevent recovery, is fever, which is either partial or univerſal. Of the latter, the ephemeris is the moſt frequent; and on that account, the author thinks, ſhould be called the puerperal fever. This ſometimes extends itſelf beyond the term of the ephemeris, and then puts puts on the appearance of inflammatory or ſlow nervous fever, according to the conſtitution of the patient. Of partial fever, the moſt alarming we are told is *peritonitis*, which is diſtinguiſhed into ſimple and complex. In the former, the inflammation attacks only the peritonæum; in the latter, it extends to one or more of the abdominal viſcera. The author obſerves, that this diſeaſe, which generally appears in child-bed, and is always attended with pyrexia, is what is commonly, but improperly called puerperal fever, as not only women in the pregnant ſtate, but men alſo are liable to it, of both which he has ſeen ſeveral inſtances. He even goes ſo far as to aſſert, " that he has not found " a diſeaſe, of equal apparent danger, nearly " ſo obedient to the laws of medicine as this." He aſſures us, that all dangerous ſymptoms have almoſt conſtantly vaniſhed upon the early and repeated uſe of an infuſion of chamomile flowers, and of a ſaline mixture, in which Rochelle ſalt and emetic tartar were diſſolved. Bleeding and ſtronger vomits he has almoſt invariably found injurious, and nitre of no material efficacy. Under the head of " natural " recovery of the infant," by which the author underſtands " its tranſition from the fœtal to

" the

" the infantile ſtate without interruption from " diſeaſe," he offers ſome few remarks on the management of children; after which he cloſes his work with a conciſe deſcription of their diſeaſes.

